# Primary Hepatic Neuroblastoma: A Case Report of a Solitary Liver Mass in an Adolescent Male

**DOI:** 10.7759/cureus.117304

**Published:** 2026-10-01

**Authors:** Mais Jazazi, Ruba Alhazaimeh, Ayman Alhwayan, Mohammed Alsmadi, Atwa Altawarh, Suliman Aljaafreh, Ruba Y Shannaq, Khaled Alshar, Maher Khader

**Affiliations:** 1 Department of Paediatric Haematology-Oncology, Royal Medical Services - Queen Rania Children’s Hospital, Amman, JOR; 2 Department of Paediatric Radiology, Royal Medical Services - Queen Rania Children’s Hospital, Amman, JOR; 3 Department of Pathology and Laboratory Medicine, Royal Medical Services - Princess Iman Center for Research and Laboratory, Amman, JOR; 4 Department of Paediatric Surgery, Royal Medical Services - Queen Rania Children’s Hospital, Amman, JOR

**Keywords:** adolescent, neuroblastoma, pediatric liver tumor, primary hepatic neuroblastoma, solitary liver mass

## Abstract

Primary hepatic neuroblastoma (PHN) is an exceptionally rare subtype of neuroblastoma. Neuroblastoma typically arises in tissues of the sympathetic nervous system, and hepatic metastases are more common than PHN. We present a 12-year-old male who presented with abdominal discomfort. Imaging revealed hepatomegaly with a solitary mass in segment V of the liver. After total resection, histopathological analysis identified a poorly differentiated neuroblastoma with a low mitotic-karyorrhectic index (<2%) and absence of neuroblastoma (MYCN) gene amplification (International Neuroblastoma Staging System, stage 1).

This case is notable for the patient's age, successful surgical outcome, and mass confinement of the liver. Despite poor cellular differentiation yielding an unfavorable histological grade, the localized Stage 1 disease and absence of MYCN amplification supported an individualized approach resulting in the absence of disease recurrence for 18 months postoperatively without chemotherapy. PHN should be considered in the differential diagnosis for children presenting with a solitary liver mass, regardless of age.

## Introduction

Neuroblastoma is the most common extracranial solid malignancy in children and arises from embryonal neuroectodermal cells. It is most often found in the adrenal medulla (35-50% of cases) or paravertebral sympathetic ganglia [1]. The estimated annual incidence is 10.2 per million children under 15 in the United States and 65 per million in infants under one year [1]. Hepatic neuroblastoma usually represents metastasis rather than a primary tumor [2].

Primary hepatic neuroblastoma (PHN) is defined as a solitary liver mass without evidence of neuroblastoma in the sympathetic ganglia, adrenal glands, or other typical sites. Diagnosing PHN is essentially a diagnosis of exclusion; it strictly requires the careful and rigorous exclusion of an occult extrahepatic primary tumor. It is extremely rare, with few well-documented cases in the literature, and has predominantly been reported in infants and very young children under two years of age [2,3]. Because of this, PHN is challenging to diagnose and can closely resemble hepatoblastoma or metastatic neuroblastoma.

Diagnosing PHN requires excluding extrahepatic tumors through comprehensive staging and imaging, including computed tomography (CT) or magnetic resonance imaging (MRI), a metaiodobenzylguanidine (MIBG) scan, bone marrow studies, and confirmation of neural origin by histopathology [4]. Treatment depends heavily on the current risk-stratification framework for neuroblastoma, which is multifactorial. Rather than relying on a single metric, risk classification depends on a combination of the patient's age at diagnosis, disease stage (e.g., International Neuroblastoma Staging System (INSS)), tumor histology, and molecular markers.

For example, while MYCN gene amplification is present in roughly 20% of neuroblastomas and strongly indicates aggressive, high-risk disease [5], its absence, when combined with a low tumor stage and complete surgical resection, can place a patient in a low-risk category under frameworks such as the Children's Oncology Group (COG) risk stratification, sometimes allowing for observation alone even if the age-dependent histological grading is unfavorable [6]. Consequently, completely resected, localized tumors lacking high-risk molecular features may be managed with surgery alone, whereas high-risk tumors require intensive chemotherapy, stem cell rescue, and radiation [6].

We report a 12-year-old male who presented with abdominal pain due to hepatomegaly with a mass in liver segment V. Complete excision revealed a poorly differentiated neuroblastoma. This case highlights PHN diagnostic criteria and demonstrates successful surgical management in an unusually older adolescent patient. It highlights how localized disease and non-amplified MYCN status can be balanced against unfavorable histology in multidisciplinary decision-making, contributing to the limited evidence base for treating this rare condition.

## Case presentation

A previously healthy 12-year-old male presented to the emergency department with a three-week history of increased pallor, dizziness, upper abdominal pain, loss of appetite, and one episode of coffee-ground vomiting. He had no fever, significant weight loss, jaundice, rash, bone pain, or changes in bowel habits. Family history included sickle cell trait (mother) and β-thalassemia trait (father), with no history of neoplastic disease. On examination, he appeared pale with marked conjunctival pallor. The liver was firm, non-tender, and palpable 4 cm below the right costal margin. There was no splenomegaly, lymphadenopathy, or palpable abdominal mass.

Vital signs were within normal limits: temperature 36.8°C, blood pressure 108/68 mmHg, heart rate 92 bpm, and respiratory rate 18. Cardiac and pulmonary examinations were unremarkable. The liver was palpable 4 cm below the right costal margin, smooth, firm, and non-tender. There were no signs of peritoneal irritation or ascites. Neurological examination was normal, with no evidence of Horner syndrome, abnormal eye movements, or focal deficits. Skin examination revealed no nodules, raccoon eyes, or blueberry muffin spots.

Initial laboratory evaluation (Table 1) revealed hypochromic microcytic anemia (hemoglobin 6.5 g/dL) and sickle cell trait (hemoglobin S (HbS) 27%). A complete blood count (CBC) showed normal white blood cells (WBCs) and platelet counts. All other laboratory results were within normal limits. Abdominal ultrasound identified a 26 × 26 mm heterogeneous, hypoechogenic mass in liver segment V, with no evidence of vascular invasion or bile duct dilation.

**Table 1:** The patient's laboratory test results at admission, along with age- and sex-matched reference ranges for a 12-year-old male.

Test	Result	Reference Range
Complete blood count (CBC)		
White blood cells (WBC, 10^9^/L)	6.2	4.5-13.5
Hemoglobin (Hb, g/dL)	6.5	11.5-15.5
Packed cell volume (PCV, %)	22.4	35-45
Mean corpuscular volume (MCV, fL)	58.1	77-95
Platelets (PLT, 10^9^/L)	321	150-450
Absolute neutrophil count (ANC, 10^9^/L)	2.6	1.8-8.0
Absolute lymphocyte count (ALC, 10^9^/L)	3.1	1.5-6.5
Monocyte count (10^9^/L)	0.72	0.2-1.0
Kidney function tests (KFT)		
Creatinine (mg/dL)	0.41	0.3-0.7
Blood urea nitrogen (BUN, mg/dL)	14	7-18
Phosphorus (mg/dL)	5.1	3.5-5.5
Uric acid (mg/dL)	5.1	3.5-7.2
Potassium (mmol/L)	3.8	3.5-5.0
Sodium (mmol/L)	133	135-145
Calcium (mg/dL)	8.6	8.8-10.8
Liver function tests (LFT)		
Aspartate aminotransferase (AST, U/L)	18.6	10-40
Alanine aminotransferase (ALT, U/L)	7.5	10-35
Total bilirubin (mg/dL)	0.26	<1.0
Albumin (g/L)	37.1	35-50
Coagulation profile		
Prothrombin time (PT, second)	14.5	11-15
Partial thromboplastin Time (PTT, second)	28.1	25-35
International Normalized Ratio (INR)	1.12	0.8-1.2
Other Tests		
Erythrocyte sedimentation rate (ESR, mm/hr)	16	<20
Lactate dehydrogenase (LDH, U/L)	176	110-295
C-reactive protein (CRP, mg/dL)	0.6	<1.0
Beta-human chorionic gonadotropin ($\beta$-HCG, IU/L)	0.25	<5.0
Alpha-fetoprotein (AFP, ng/mL)	1.24	<10
Hemoglobin S %	27	Absent

Regarding the patient's presentation with severe hypochromic microcytic anemia (Hb 6.5 g/dL, MCV 58.1 fL) and an episode of coffee-ground vomiting, the clinical team evaluated these findings in the context of combined iron deficiency and his parental hemoglobinopathy traits (sickle cell and β-thalassemia traits). The single episode of coffee-ground vomiting was managed conservatively with intravenous proton pump inhibitors and a packed red blood cell transfusion, with no recurrence of gastrointestinal bleeding; formal upper gastrointestinal endoscopy was not pursued due to rapid clinical stabilization. Of note, plasma or urinary catecholamine metabolites (such as vanillylmandelic acid (VMA) and homovanillic acid (HVA)) were not measured during the initial workup, as the diagnosis was established via cross-sectional imaging, core needle biopsy, and subsequent surgical excision.

Liver MRI revealed an oval lesion in segment V measuring 26 by 26 mm. The lesion was hyperintense on T2-weighted imaging, with a central cystic component and a hypointense rim (Figure 1). Mild, uneven enhancement was noted. No vascular invasion or extrahepatic spread was seen. Differential diagnoses included primary liver malignancy, hepatoblastoma, metastatic lesion, or hemangioma. Ultrasound-guided biopsy identified a blue cell malignancy, with possibilities including neuroendocrine tumor, neuroblastoma, and melanoma. As tumor markers were normal and the biopsy was inconclusive, a surgical consult was done, and they decided to excise the tumor.

**Figure 1:**
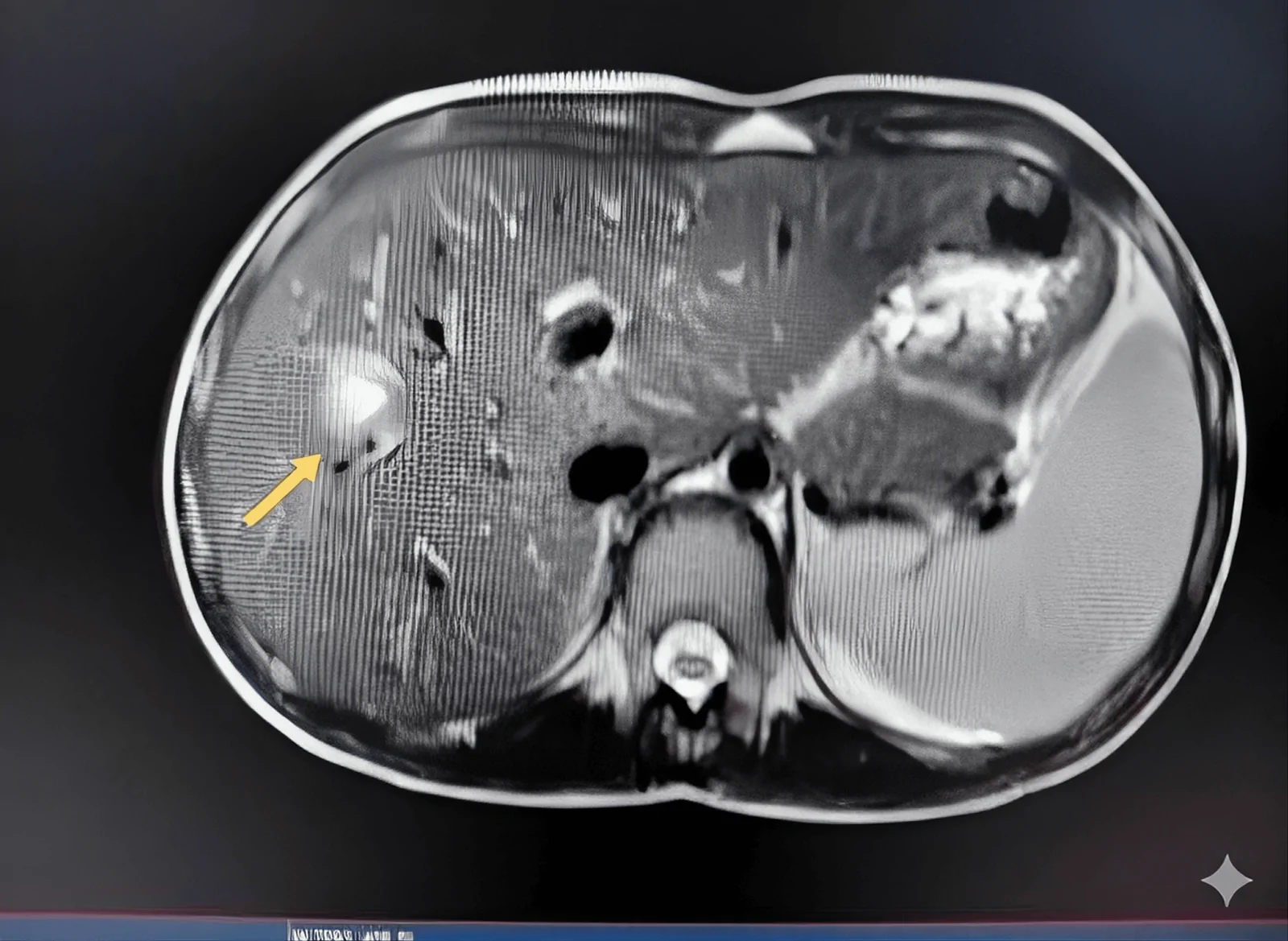
T2-weighted abdominal MRI showing the lesion located in segment V of the liver. The oval mass measures approximately 26 × 26 mm and is characterized by central hyperintensity (cystic/necrotic component) surrounded by a distinct hypointense rim. (Readers can locate this lesion in the lower right/anterior portion of the liver parenchyma as displayed in the axial view).

The excised tumor in segment V was well delineated, measuring 2.6 by 2.6 by 2.3 cm, and appeared white, rubbery, and firm, with focal hemorrhage and central cavitation. Histological examination (Figure 2) showed small round blue cells with rosettes and neutrophils and a low mitotic-karyorrhectic index (MKI) of less than 2% in 5,000 cells. Ten lymph nodes and the gallbladder were also excised, with no evidence of malignancy, and peritoneal fluid was negative for cancer cells. Surgical pathology confirmed complete excision with clear microscopic margins (R0 resection). The tumor stained positive for synaptophysin, Cluster of Differentiation (CD) 56, and neuron-specific enolase (NSE), with no MYCN amplification detected by Fluorescence In Situ Hybridization (FISH). (Note: Extended molecular cytogenetics such as tumor ploidy, DNA index, and 11q segmental chromosomal status, as well as PHOX2B and chromogranin A immunostaining, were not performed due to diagnostic panel limitations at our center).

**Figure 2:**
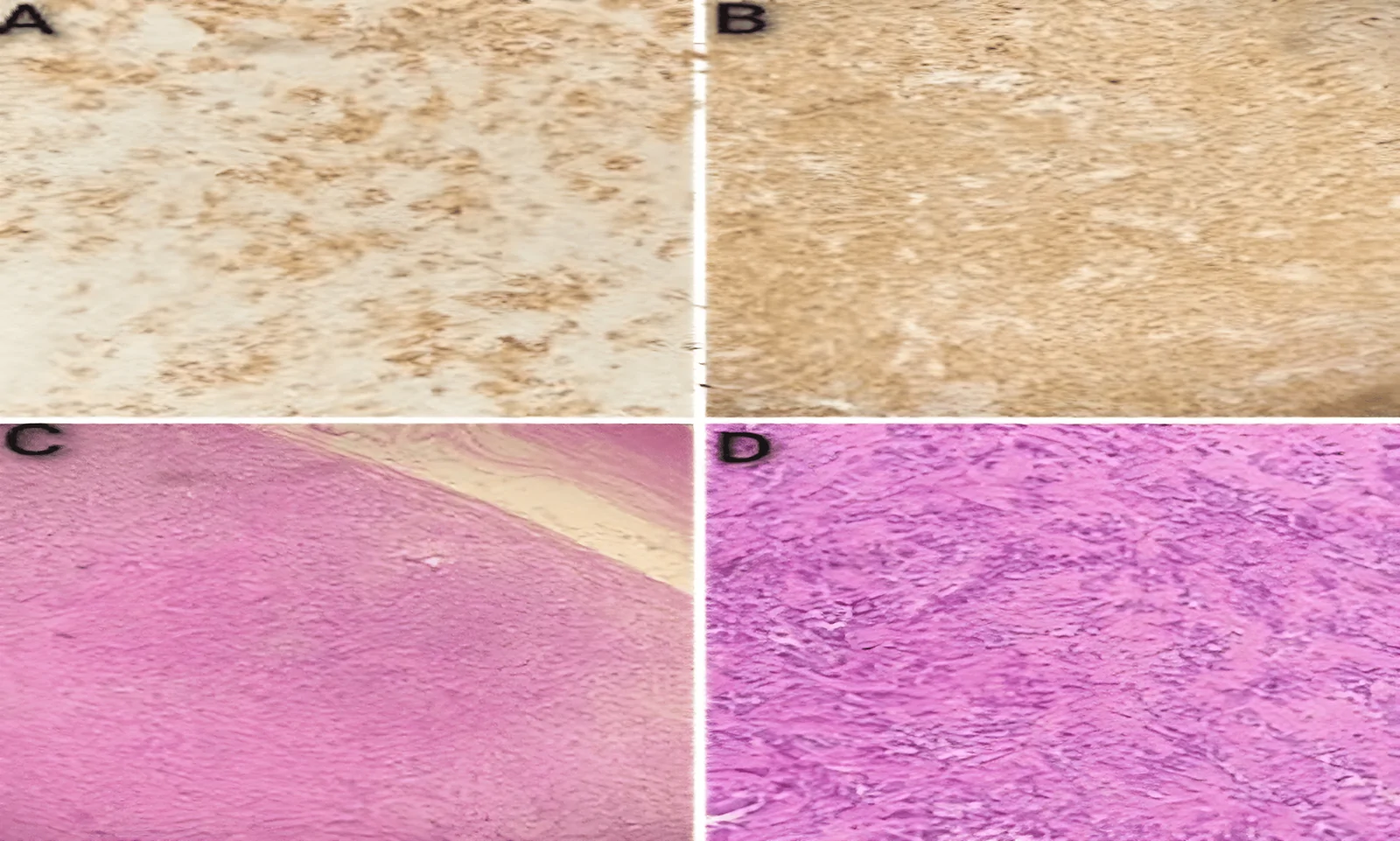
Histopathological and immunohistochemical analysis of the liver mass. (A) Tumor cells positive for synaptophysin immunostain (synaptophysin stain, 200× magnification). (B) Tumor cells positive for neuron-specific enolase (NSE) immunostain (NSE stain, 200× magnification). (C) Low-power view of a well-defined nodule of poorly differentiated neuroblastoma with a residual hepatic rim at the top right (Hematoxylin and Eosin (H&E) stain, 50× magnification). (D) High-power view showing a cellular tumor of uniform round cells with hyperchromatic nuclei, scant eosinophilic cytoplasm, indistinct borders, and focal pseudorosettes (H&E stain, 200× magnification).

According to the International Neuroblastoma Staging System (INSS) [7], the patient was classified as Stage 1, characterized by a localized tumor with complete gross and microscopic resection and negative regional lymph nodes. Separately, histological evaluation was interpreted within the framework of the International Neuroblastoma Pathology Classification (INPC). As the patient was older than 18 months, the INPC graded the tumor as having pathologically unfavorable histology, despite its low MKI. The patient underwent extensive evaluation to check for disease elsewhere. The evaluation showed a normal bone marrow biopsy, a negative technetium-99m bone scan, and no disease on chest, abdomen, and pelvic CT scans, as well as no MIBG-avid lesions on the I-123 MIBG scan.

The pediatric hematology-oncology team opted for observation without chemotherapy, based on the patient's INSS Stage 1 classification, complete tumor resection, low MKI, and absence of MYCN amplification. This approach differs from protocols for high-risk neuroblastoma. Postoperative surveillance included monthly abdominal ultrasounds and clinical evaluations for the first three months, followed by bi-monthly assessments for the subsequent nine months, and continued every three to six months thereafter. Routine liver function tests and CBTs remained consistently normal. At the 18-month postoperative mark, the patient remained completely disease-free and had resumed normal activities without adverse effects, and follow-up continues according to low-risk INSS guidelines.

## Discussion

PHN is an exceptionally rare pediatric malignancy, comprising only a handful of well-documented cases in the literature [2,3]. While classic neuroblastoma commonly arises in the adrenal medulla or paraspinal sympathetic ganglia and subsequently metastasizes to the liver, PHN presents as a solitary hepatic mass in the complete absence of an extrahepatic primary site [8]. In this case, comprehensive staging, including normal cross-sectional imaging of the adrenal glands and sympathetic chain, a normal bone marrow biopsy, and a negative MIBG scan, successfully fulfilled the diagnostic criteria for PHN while ruling out metastatic disease.

Diagnosing PHN remains challenging because its clinical and radiological features frequently overlap with other pediatric small round blue cell tumors, such as hepatoblastoma, yolk sac tumor, or mesenchymal malignancies. Histopathological and immunohistochemical confirmation are therefore critical. In our patient, strong immunoreactivity for synaptophysin, CD56, and NSE confirmed the neural origin of the tumor. However, we acknowledge as a diagnostic limitation that extended immunohistochemical markers (such as PHOX2B or chromogranin A) and secondary biochemical or molecular assays, specifically urinary or plasma catecholamine metabolites (VMA/HVA) and advanced cytogenetics (tumor ploidy and 11q chromosomal status), were not evaluated due to initial institutional panel constraints. Nonetheless, the integration of core needle biopsy, definitive surgical pathology, and negative metastatic workup secured the diagnosis.

The demographic profile of our patient introduces an unusual clinical aspect. PHN predominantly affects infants and toddlers under two years of age [2,9]. Published literature features successful surgical and medical interventions in infant cohorts [2], alongside instances of aggressive clinical courses in young children [9]. Furthermore, Jung published a well-documented case of PHN successfully managed with surgical resection [10]. To the best of our knowledge, our patient represents a rare instance of PHN in an adolescent managed with surgery alone without subsequent recurrence at intermediate follow-up.

Managing localized neuroblastoma relies on a complex, multifactorial risk-stratification framework rather than any single metric. Contemporary protocols weigh patient age, disease stage, tumor histology, and molecular aberrations [6,11]. In our case, the multidisciplinary decision to pursue observation rather than adjuvant chemotherapy was highly individualized, balancing both favorable and unfavorable disease features. Specifically, the patient presented with INSS Stage 1 disease (R0 resection) and non-amplified MYCN. Under the COG risk stratification framework, INSS Stage 1 tumors lacking MYCN amplification are classified as low risk, which generally permits observation. While the patient's adolescent age and poorly differentiated histology resulted in an unfavorable INPC classification, which is traditionally an adverse prognostic indicator [11], the localized stage and absence of MYCN amplification justified withholding multi-agent chemotherapy [12].

This individualized approach was undertaken with the understanding that our institutional inability to evaluate extended molecular markers (such as tumor ploidy or 11q status) or biochemical markers (VMA/HVA) introduced some uncertainty into the exact risk profile, thereby mandating rigorous postoperative surveillance. This aligns with risk-adapted principles, avoiding overtreatment and its associated late morbidity in indolent or completely resected presentations [13], though no standardized treatment consensus currently exists specifically for adolescent PHN [14].

Given that neuroblastoma carries a recognized risk of late recurrence, even years after initial therapy in low-risk cohorts [13,15], rigorous long-term surveillance is mandatory. Our patient has maintained an 18-month disease-free interval with normal liver function and clinical well-being. Ultimately, this case demonstrates that PHN can occasionally present in adolescents and that complete surgical resection, combined with risk-adapted observation, can yield excellent outcomes in localized, non-amplified MYCN disease. Additional multicenter registries and case documentation remain essential to refine management guidelines and long-term prognostic markers for this rare entity.

Limitations

Despite the clinical value of this case report, several limitations should be acknowledged. First, institutional assay availability at the time: extended molecular and cytogenetic evaluations, such as tumor ploidy, DNA index, and 11q segmental chromosomal aberrations, were not performed. Second, urinary or plasma catecholamine metabolites (such as VMA and HVA), which are classically evaluated in neuroblastoma workups, were not quantitatively measured, as the diagnosis was established via cross-sectional imaging, core needle biopsy, and definitive surgical pathology. Finally, as a single-case report from a single center, our findings, though supported by an 18-month disease-free interval, highlight the need for multi-center registries to establish standardized, evidence-based guidelines specifically tailored for adolescent PHN.

## Conclusions

PHN is an exceptionally rare pediatric malignancy that presents unique diagnostic challenges. This case describes an unusual presentation of a localized PHN in a 12-year-old adolescent. While the patient's age and poor cellular differentiation yielded an unfavorable histological grading under the INPC framework, the complete surgical resection (R0) of Stage 1 disease, low mitotic-karyorrhectic index (<2%), and absence of MYCN amplification collectively supported a highly individualized multidisciplinary decision to omit adjuvant chemotherapy. Given the single-case nature of this report, the limited 18-month disease-free follow-up, and the known risk of late neuroblastoma relapse, these findings do not establish a broad recommendation or universal treatment standard for adolescent PHN. Rather, they demonstrate the feasibility of an individualized, risk-adapted surgical and observational approach in an exceptionally rare clinical scenario where favorable clinical and molecular features coexist with unfavorable histology. Comprehensive staging, multidisciplinary risk assessment, and prolonged, rigorous long-term surveillance remain essential.
